# L-(−)-(*N*-*trans*-Cinnamoyl)-arginine, an Acylamino Acid from *Glinus oppositifolius* (L.) Aug. DC.

**DOI:** 10.3390/molecules15096186

**Published:** 2010-09-02

**Authors:** Poolsak Sahakitpichan, Wannaporn Disadee, Somsak Ruchirawat, Tripetch Kanchanapoom

**Affiliations:** 1 Chulabhorn Research Institute and Chulabhorn Graduate Institute, Vipavadee-Rangsit Highway, Bangkok 10210, Thailand; E-Mails: poolsak@cri.or.th (P.S.); wannapor@cri.or.th (W.D.); 2 The Center of Excellence on Environmental Health, Toxicology and Management of Chemicals, Vipavadee-Rangsit Highway, Bangkok 10210, Thailand; E-Mail: somsak@cri.or.th; 3 Faculty of Pharmaceutical Sciences, Khon Kaen University, Khon Kaen 40002, Thailand

**Keywords:** *Glinus oppositifolius*, Molluginaceae, L-(−)-(*N*-*trans*-cinnamoyl)-arginine, arginine derivative, flavonol glycoside, flavone *C*-glycoside

## Abstract

An amino acid derivative, L-(−)-(*N*-*trans*-cinnamoyl)-arginine, was isolated from the whole plant of *Glinus oppositifolius* (L.) Aug. DC. along with kaempferol 3-*O*-galactopyranoside, isorhamnetin 3-*O*-β-D-xylopyranosyl-(1→2)-β-D-galactopyranoside, vitexin, vicenin-2, adenosine and L-phenylalanine. The structure determinations were based on analyses of chemical and spectroscopic methods.

## 1. Introduction

*Glinus oppositifolius* (L.) Aug. DC. (Mollungincaeae; Thai name: Phak-Khee-Khuang) is an annual prostrate weed commonly found in paddy fields after harvesting, especially in the Northeast areas of Thailand. The leaves are used as vegetable for cooking purposes, as well as an expectorant and antipyretic agent. This species has been also found in other tropical parts of Asia, Africa and North Australia. Plants in this genus are reported to contain triterpenoidal saponins [1,2,3,4,5,6]. In previous studies of this plant, triterpenoidal saponins and pectin polysaccharides were reported to act as antiprotozoal [6] and immunomodulating agents [7,8], respectively. The plant extracts showed antifungal, larvicidal, molluscicidal, free scavenging and antioxidant activities [9,10].

The present paper deals with the isolation and characterization of a new amino acid derivative **1** from *G. oppositifolius*, together with six known compounds, including four flavonoid glycosides **2–5**, one nucleoside **6**, and one amino acid **7**, from the water soluble fraction of the whole plant.

## 2. Results and Discussion

The methanolic extract of *G. oppositifolius* was suspended in H_2_O and partitioned with Et_2_O. The aqueous layer was separated by the combinations of Diaion HP20 column, silica gel column, RP-18 column and preparative HPLC-ODS column chromatography to provide seven compounds **1–7**. 

**Figure 1:**
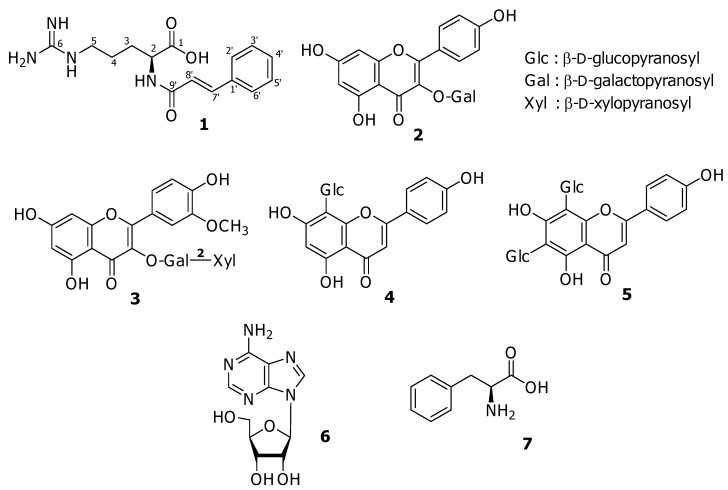
Structures of isolated compounds **1–7.**

**Figure 2:**
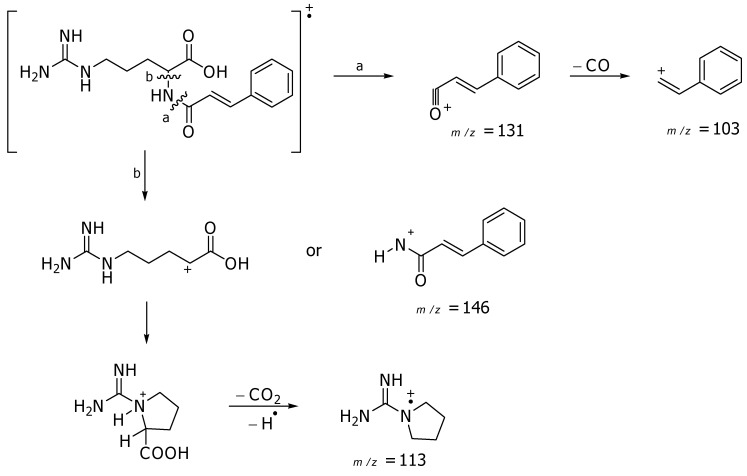
Possible major fragment ions of compound **1**.

Compound **1** was isolated as an amorphous powder. Its molecular formula was determined as C_15_H_20_N_4_O_3_ by high-resolution electrospray ionization time-of-flight (ESITOF) mass spectrometric analysis. Inspection of the IR spectrum indicated the presence of the typical amine N-H stretching absorption band at 3333 and 3165 cm^−1^. The band at 1667 cm^−1^ is a characteristic for the C=O stretching of an amide and a carboxylic acid, 1613 cm^−1^ for the C=N of a quanidyl group, and the 1571 cm^−1^ one for amine group bending vibrations [11,12]. The ^1^H-NMR spectrum exhibited a *trans*-cinnamoyl moiety inferred from a set of five protons corresponding to an aromatic ring at δ_H_ 7.32 (2H), 7.35 (1H) and 7.43 (2H), in addition to two olefin protons at δ_H_ 6.74 and 7.28 (each *d*). The *trans* configuration was assigned from the coupling constant with *J* = 15.8 Hz. The ^13^C-NMR spectrum showed 13 carbon signals (Table 1), of which nine were assignable to a *trans*-cinnamoyl moiety. The remaining carbon signals belong to three methylenes (δ_C_ 25.5, 30.2, 40.8), one methine (δ_C_ 54.3) and two quaternary carbons (δ_C_ 157.7, 176.6), compatible to those of the amino acid arginine [13]. Therefore, this compound was suggested to be a *trans*-cinnamoyl derivative of arginine. The complete assignments were established by analyzing the 2D-NMR spectra including COSY, HSQC and HMBC. In the HMBC spectrum (Table 1), the significant correlations were observed between δ_H_ 4.13 (H-2) and δ_C_ 164.7 (C-9’), indicating that one of the hydrogen atoms of the α-amino group was replaced by the cinnamoyl moiety. In order to determine the absolute configuration, L-(−)-(*N*-*trans*-cinnamoyl)-arginine (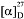 − 15.8° in 6 N HCl) was synthesized by acylation of L-(+)-arginine (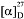 + 24.5° in 6 N HCl) with *trans*-cinnamoyl chloride (see Experimental) [12] to compare the optical rotation value. Since the isolated compound showed the optical rotation value at 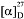 − 14.9° in 6 N HCl, the absolute configuration at C-2 position was concluded to be (*S*). Consequently, compound **1** was identical to the synthetic compound, L-(−)-(*N*-*trans*-cinnamoyl)-arginine. Furthermore, the electron ionization mass spectrometric analysis showed the fragment ions of this compound at *m/z* 131 (100), 146 (59), 103 (45), 113 (35) as illustrated in Figure 2. The structure of this compound was initially proposed in 1986 as a synthetic compound for exploration of carboxypeptidase N functions [14]. The physical and spectroscopic data were given here, and it should be noted that this compound has been reported from the natural sources for the first time.

**Table 1:** NMR spectroscopic data of compound **1** (in DMSO-*d*_6_, ^13^C-NMR: 100 MHz and^ 1^H-NMR: 400 MHz).

Position	δ_C_	δ_H_	HMBC
*Amino acid*			
1	176.6		
2	54.3	4.13 (1H, *m*)	1, 3, 4, 9'
3	30.2	1.62 (1H, *m*)	1, 2, 4, 5
		1.75 (1H, *m*)	1, 2, 4, 5
4	25.5	1.48 (2H, *m*)	2, 3, 5
5	40.8	3.05 (2H, *m*)	3, 4, 6
6	157.7		
2-NH		8.21 (1H, *d*, *J* = 8.0 Hz)	1, 2, 3, 8', 9'
*Cinnamoyl moiety*			
1'	135.5		
2', 5'	127.8	7.43 (2H, *dd*, *J* = 8.0, 1.7 Hz)	7'
3', 6'	129.2	7.32 (2H, *m*)	1'
4'	129.6	7.35 (1H, *m*)	
7'	138.4	7.28 (1H, *d*, *J* = 15.8 Hz)	1', 2', 8', 9'
8'	123.3	6.74 (1H, *d*, *J* = 15.8 Hz)	1', 7', 9'
9'	164.7		

Compounds **2** and **3** were identified as kaempferol 3-*O*-β-D-galactopyranoside and isorhamnetin 3-*O*-β-D-xylopyranosyl-(1→2)-β-D-galactopyranoside, respectively. [15,16] The NMR spectroscopic data of compounds **4** and **5** were coincident with those of apigenin 8-*C*-β-D-glucopyranoside (vitexin) and apigenin 6,8-di-*C*-β-D-glucopyranoside (vicenin-2), respectively. [17,18] Compounds **6** and **7** were assigned to adenosine and L-phenylalanine, respectively, by comparison of physical and NMR spectral data with authentic samples [19].

## 3. Experimental

### 3.1. General

^1^H- and ^13^C-NMR spectra were recorded in DMSO-*d*_6_ using a Bruker AV-400 (400 MHz for ^1^H-NMR and 100 MHz for ^13^C-NMR) or a Varian Gemini 2000 (200 MHz for ^1^H-NMR and 50 MHz for ^13^C-NMR) spectrometer. IR spectra were obtained from a Perkin Elmer Universal FTIR spectrometer. Low resolution mass spectrometry (LRMS) was performed using a Finnigan Polaris mass spectrometer. High resolution mass spectrometry (HRMS) was performed using a Bruker Micro TOF-LC mass spectrometer. Optical rotations were measured with a Jasco P-1020 digital polarimeter. For column chromatography, Diaion HP-20 (Mitsubishi Chemical Industries Co. Ltd.), silica gel 60 (70-230 mesh, Merck), and RP-18 (50 μm, YMC) were used. HPLC was carried out on an ODS column (20 X 250 mm i.d., YMC) with a Jasco RI-2031 refractive index detector. The flow rate was 6 mL/min. The solvent systems were: I) EtOAc-MeOH (9:1); II) EtOAc-MeOH-H_2_O (40:10:1); III) EtOAc-MeOH-H_2_O (70:30:3); IV) EtOAc-MeOH-H_2_O (6:4:1); V) 10−80% aqueous MeOH; VI) 2% aqueous MeCN; VII) 5% aqueous MeCN; VIII) 15% aqueous MeCN; and IX) 20% aqueous MeCN. The spraying reagent used for TLC was 10% H_2_SO_4_ in 50% EtOH.

### 3.2. Plant Material

*Glinus oppositifolius* (L.) Aug. DC. was collected in January 2008 from Khon Kaen province, Thailand. The plant was identified by Mr. Nopporn Nontapa of Department of Pharmaceutical Botany and Pharmacognosy, Faculty of Pharmaceutical Sciences, Khon Kaen University. A voucher specimen (TK-PSKKU-0063) has been deposited in the Herbarium of the Faculty of Pharmaceutical Sciences, Khon Kaen University.

### 3.3. Extraction and Isolation

Dried whole *Glinus oppositifolius* (1.4 kg) was extracted three times with CH_3_OH (8 L for each extraction) at room temperature. The solvent was concentrated *in vacuo* to give a greenish powder (226.4 g). This residue was suspended in H_2_O and defatted with Et_2_O three times (1 L each). The aqueous soluble fraction (113.6 g) was applied to a column of Diaion HP-20 and eluted successively with H_2_O, CH_3_OH, and (CH_3_)_2_CO. The fraction eluted with CH_3_OH (20.0 g) was concentrated to dryness and subjected to a silica gel column using solvent systems I (4.0 l), II (4.0 l), III (6.0 l) and IV (5.0 l) providing six fractions (fraction A−F). Fraction B (2.6 g) was applied to a column of RP-18 using solvent system V to give 12 fractions. Fraction B-8 was purified by preparative HPLC-ODS with solvent system IX to afford compound **2** (10.9 mg). Fraction B-11 was purified by preparative HPLC-ODS with solvent system IX to give compound **4** (73.1 mg). Fraction C (3.2 g) was separated on a column of RP-18 using solvent system V to give nine fractions. Compound **6** (40.1 mg) was obtained from fraction C-3 by crystallization. Fraction D (5.0 g) was subjected to a column of RP-18 using solvent system V to give 12 fractions. Fractions D-3 and D-4 were combined and purified by preparative HPLC-ODS with solvent system VIII to afford compound **5** (317.3 mg). Fraction D-9 was further purified by preparative HPLC-ODS to yield compound **3** (13.0 mg). Fraction E (3.4 g) was chromatographed on a column of RP-18 using solvent system V to provide six fractions. Fraction E-1 was purified by preparative HPLC-ODS with solvent system IX to give compound **7** (31.7 mg). Finally, fraction E-4 was purified by preparative HPLC-ODS with solvent system VIII to obtain compound **1** (115.0 mg).

### 3.4. L-(−)-(N-trans-cinnamoyl)-Arginine ***(1)***.

Amorphous powder: 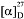 − 14.9° (6 N HCl, *c* 0.22); IR (UATR) *ν*_max_ 3333, 3165, 3047, 1667, 1613, 1571, 1400, 1245, 979 cm^−1^; ^1^H- and ^13^C-NMR (DMSO-*d*_6_): Table 1; EIMS: *m/z* 131 (100), 146 (59), 103 (45), 113 (35); Negative HRMS(ESITOF), *m/z*: 303.1468 [M − H]^–^ (calcd for C_15_H_19_N_4_O_3_, 303.1463).

### 3.5. Synthesis of L-(−)-(N-trans-cinnamoyl)-Arginine ***(1)***

L-(+)-Arginine (50.2 mg) was dissolved in H_2_O (2.0 mL) and the pH adjusted to 12.0 with 2 N NaOH. A solution of *trans*-cinnamoyl chloride (51.5 mg) in dioxane (2.0 mL) was added. After stirring at room temperature for 30 min, the reaction was concentrated and purified by preparative HPLC-ODS with solvent system VIII to obtain L-(−)-(*N*-trans-cinnamoyl)-arginine (15.0 mg) as an amorphous powder, 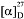 − 15.8° (6 N HCl, *c* 0.12). The NMR spectroscopic data was identical to those of the natural compound.

## 4. Conclusions

Isolated compounds of Thai plant origin were described as an acylamino acid **1**, flavonoid glycosides **2–5**, a nucleoside **6**, and an amino acid **7**. The results of our study were different from the previous mention on the chemical constituents of genus *Glinus*, indicating triterpenoidal saponins. The chemical variations might be due to the different geographical regions involved. The study identified new types of compounds in this species.
